# Oxygen availability influences the incidence of testicular teratoma in *Dnd1Ter/+* mice

**DOI:** 10.3389/fgene.2023.1179256

**Published:** 2023-04-26

**Authors:** Ximena M. Bustamante-Marin, Blanche Capel

**Affiliations:** ^1^ Department of Cell Biology, Duke University Medical Center, Durham, NC, United States; ^2^ Departamento Biomédico, Facultad de Ciencias De La Salud, Universidad de Antofagasta, Antofagasta, Chile

**Keywords:** testicular teratomas, hypoxia, nodal signaling, TER, DND1

## Abstract

Testicular teratomas and teratocarcinomas are the most common testicular germ cell tumors in early childhood and young men, and they are frequently found unilaterally in the left testis. In 129/SvJ mice carrying a heterozygous copy of the potent modifier of tumor incidence *Ter*, a point mutation in the dead-end homolog one gene (*Dnd1* ^*Ter/+*^), ∼70% of the unilateral teratomas arise in the left testis. We previously showed that in mice, left/right differences in vascular architecture are associated with reduced hemoglobin saturation and increased levels of the hypoxia inducible factor-1 alpha (HIF-1α) in the left compared to the right testis. To test the hypothesis that systemic reduction of oxygen availability in *Dnd1* ^*Ter/+*^ mice would lead to an increased incidence of bilateral tumors, we placed pregnant females from 129/SvJ *Dnd1* ^*Ter/+*^ intercross matings in a hypobaric chamber for 12-h intervals. Our results show that in 129/SvJ *Dnd1* ^*Ter/+*^ male gonads, the incidence of bilateral teratoma increased from 3.3% to 64% when fetuses were exposed to acute low oxygen conditions for 12-h between E13.8 and E14.3. The increase in tumor incidence correlated with the maintenance of high expression of pluripotency genes *Oct4*, *Sox2* and *Nanog*, elevated activity of the Nodal signaling pathway, and suppression of germ cell mitotic arrest. We propose that the combination of heterozygosity for the *Ter* mutation and hypoxia causes a delay in male germ cell differentiation that promotes teratoma initiation.

## 1 Introduction

Testicular teratomas (benign, mature, and immature) and teratocarcinomas (malignant) are the most common testicular germ cell tumors found in early childhood and young men (Hussain et al., 2008; Richardson et al., 2008; Nerli et al., 2010). These tumors (together referred to as teratomas) show high histological diversity and contain a variety of tissue elements and differentiated cell types derived from all three germ layers. Originally, teratomas were difficult to study because they were rare, but in 1973, Stevens discovered a spontaneous mutation (*Ter*) that arose on the inbred mouse strain, 129/SvJ, that increased the incidence of testicular teratoma from a background of 1% to >30% in 129/SvJ males (Stevens and Little, 1954; Stevens, 1973). In the 1960s, Stevens performed a series of experiments showing that testicular teratomas originate from germ cells (Stevens, 1964). Steven’s work led to the recognition that germ cells exist as a pluripotent stem cell (capable of differentiating into most, if not all, somatic cell types) during their early development. However, how this potential is harnessed during the development of male germ cells into unipotent spermatogonial stem cells (SSCs), and what role the gene underlying the *Ter* mutation plays in this process remain unanswered questions.

In the mouse, primordial germ cells (PGCs) are specified around E6.25 from the proximal epiblast under the control of bone morphogenetic protein (BMP) signals that suppress the somatic differentiation program and promote the expression of the core transcriptions factors *Oct4*, *Sox2* and *Nanog* (Saitou et al., 2002; Ohinata et al., 2005; Yabuta et al., 2006). In all genetic backgrounds, *Ter/Ter* mice show apoptotic loss of primordial germ cells soon after specification (Noguchi et al., 1996), but in the 129/SvJ genetic background, some germ cells escape the initial wave of apoptosis and arrive in the genital ridges between E10.5 and E11.5 (Cook et al., 2009). Germ cells normally undergo epigenetic changes including global DNA demethylation and acquisition of histone marks such as H3K27me3 (Seki et al., 2007) during their migration to the gonad. Upon arrival in the gonad, the signals from the testicular somatic environment, including *Fgf9* (DiNapoli et al., 2006) and the transient upregulation of *Nodal* and *Cripto* (Spiller et al., 2012), stimulate germ cell proliferation and control the number and pluripotency of the future spermatogonia stem cell (SSC) population. Male gonocytes proliferate until they enter mitotic arrest in G1/G0. By E14.5, more than 80% of male germ cells are in G1/G0 in 129T2, CD-1, and C57BL/6 strains of mice (Western et al., 2011). Mitotic arrest in G0 involves regulation of the G1/S checkpoint, through the action of Retinoblastoma 1 (Spiller et al., 2010; Yang et al., 2013) and negative cell cycle regulators such as P27 (Western et al., 2008). Genome-wide changes, including active repression of genes associated with pluripotency, and maintenance of silencing at loci associated with somatic differentiation, occurs as cells transition to T1 prospermatogonia during mitotic arrest (Western et al., 2008; Western et al., 2011; Seisenberger et al., 2012; Hargan-Calvopina et al., 2016). It has been suggested that teratomas arise during this window of germ cell development as a result of a failure to repress genes that maintain PGC pluripotency (Noguchi and Stevens, 1982; Heaney et al., 2012; Webster et al., 2021).

In 2005, the *Ter* mutation was mapped to a point mutation that introduced a premature STOP codon in the gene *Dnd1* (*Dnd1* ^*Ter*^) (Youngren et al., 2005; Zhang et al., 2021). *Dnd1* encodes an RNA-binding protein for which several functions have been described, and multiple RNA targets have been identified (Yamaji et al., 2017; Ruthig et al., 2019; Ruthig et al., 2021). In partnership with NANOS2, DND1 associates with the CCR4-NOT complex to promote the degradation of target transcripts (Suzuki et al., 2016; Yamaji et al., 2017; Hirano et al., 2022). In addition, by binding to 3′- untranslated regions and antagonizing repression of translation mediated by microRNAs, DND1 promotes target translation, including transcripts that negatively regulate the cell cycle (Kedde et al., 2007). Two of these, *p27* and *p21*, are normally expressed at the onset of G0 in male germ cells (Western et al., 2008). In *Dnd1* ^*Ter/Ter*^ mice, *p21* and *p27* are not translated, suggesting an explanation for why male germ cells skip cell cycle arrest (Cook et al., 2011). In heterozygous *Dnd1* 129/SvJ mice (*Dnd1* ^*Ter/+*^), the incidence of both teratoma and spermatogenic failure is much lower, and typically occurs unilaterally with a strong bias to the left testis (Stevens and Little, 1954). Interestingly, crossing of the *situs inversus viscerum* mutation onto the 129/SvJ *Dnd1* ^*Ter/+*^ genetic background led to a transposition of the organs and blood vessels and the reversal of the bias to the right testis (Stevens, 1982). These results suggested that the bias of spermatogenic failure or teratoma development in 129/SvJ *Dnd1* ^*Ter/+*^ mice is due to differences that arise from body axis asymmetry. Previous results from our lab, showed that increased spermatogenic failure in the left testis of 129/SvJ *Dnd1* ^*Ter/+*^ correlated with reduced hemoglobin saturation, higher levels of hypoxia-inducible factor-1 alpha (HIF-1α), and metabolic differences, including lower levels of ATP and NADH (Bustamante-Marin et al., 2015). These results suggested that the microenvironment of the gonad affects the fate of male germ cells.

Oxygen is a key substrate for cellular metabolism and bioenergetics. Hypoxia, or decrease in oxygen tension, results in the activation of different responses at both cellular and whole organism levels. In mammals, the primary transcriptional response to hypoxic stress is mediated by HIFs (Wang et al., 1995). The response to hypoxia depends on the context in which it occurs. However, hypoxia has been shown to induce the undifferentiated state of hematopoietic, mesenchymal and neural stem cells, promoting self-renewal (Gustafsson et al., 2005; Mohyeldin et al., 2010; Park et al., 2013; Binh et al., 2014; Araki et al., 2021) and to induce pluripotency in previously committed cells (Mathieu et al., 2013). Consistent with the idea that low oxygen conditions can promote the pluripotent state of male germ cells, Oatley and co-workers found that shifting to glycolytic conditions by lowering oxygen tension from 21% to 10%, increased efficiency of SSCs maintenance *in vitro*, and improved subsequent SSCs transplantation efficiency (Helsel et al., 2017).

Based on these findings, we hypothesized that the left-bias in teratoma incidence in 129/SvJ *Dnd1* ^*Ter/+*^ mice, arising from differential oxygen availability due to differences in body vascular architecture, would be abolished by a systemic reduction of oxygen tension. To test this hypothesis, we exposed pregnant females from 129/SvJ *Dnd1* ^*Ter/+*^ intercross matings to hypoxia (∼10% oxygen). Pregnant females were placed in a hypobaric chamber for periods of 12-h on each day between E10.8 and E15.8. In male 129/SvJ *Dnd1* ^*Ter/+*^ offspring from females exposed between E13.8-E14.3, the incidence of bilateral teratoma increased from 3.3% to 64%, abolishing the asymmetric left-bias. The increase in tumor incidence correlated with high levels of HIF-1α, maintenance of high expression of pluripotency genes *Oct4*, *Sox2* and *Nanog*, elevated activity of the Nodal signal pathway, and suppression of germ cell mitotic arrest. We conclude that heterozygosity for the *Ter* mutation can be used as a sensor to detect factors in the testis microenvironment, such as low oxygen tension, that contribute to mis-regulation of male germ cell development leading to teratoma initiation.

## 2 Materials and methods

### 2.1 Mice

All animal experiments were conducted according to Duke University Medical Center-Institutional Animal Care and Use Committee and National Institutes of Health guidelines. *Dnd1* ^*Ter/+*^ *: Oct4-EGFP* (enhanced green fluorescent protein) mice were maintained on a 129/SvJ background, genotyped, and crossed as described previously (Cook et al., 2009). For timed matings, *Dnd1* ^*Ter/+*^ *: Oct4-EGFP* males and females were placed together in the afternoon, and plugs found the following morning were counted as embryonic stage (E) 0.5.

### 2.2 Hypoxia exposure

Pregnant females from timed matings between 129/SvJ *Dnd1* ^*Ter/+*^ *;Oct4-EGFP* mice were placed in a hypobaric chamber for one 12 h period (from 7 p.m. to 7 a.m.) on E10.8, E11.8, E12.8, E13.8, E14.8 or E15.8 days of development. The hypobaric chamber for animal studies, located at the Duke center for Hyperbaric medicine and Environmental Physiology, was kindly provided by Dr. Claude Piantadosi. The chamber was pressurized to simulate an altitude of (18,000 ± 1000 feet, ∼5,500 m), which equals a barometric pressure of ∼53 kPa and 10.5% effective oxygen availability (21% at sea level), leading to an estimated reduction of the partial pressure of arterial oxygen (PaO_2_) from ∼12 kPa in normoxia to ∼3.5 kPa. After the acute hypoxic exposure, pregnant females were returned to normoxic conditions and sacrificed on days E14.5- E18.5. The embryos were recovered, the male gonads were dissected and dissociated for fluorescence-activated cell sorting (FACS) to isolate Oct4:GFP^+^ germ cells. Alternatively, gonads were collected for whole mount immunofluorescence for the screening of teratoma incidence based on E-Cadherin staining.

### 2.3 Cryo-immunofluorescence

Fluorescent immunocytochemistry was performed in frozen sections of the right and left male gonads dissected from controls and after exposure to hypoxia. Gonads from *Dnd1* ^*+/+*^ and *Dnd1* ^*Ter/+*^ male mice expressing the OCT4-EGFP reporter at stages E14.5 and E18.5 were dissected in phosphate-buffered saline (PBS), fixed in 4% paraformaldehyde (PFA) (Thermo Fisher Scientific) overnight at 4°C and processed for cryo-sectioning. Immunostaining was performed as described previously (Bustamante-Marin et al., 2015). Primary antibodies and dilutions used are listed in Supplementary Table S1. Fluorescent secondary Cy5-or Cy3-conjugated (1:500 dilution; Jackson ImmunoResearch) or Alexa 647- or Alexa 488-conjugated (1:500 dilution; Molecular Probes Inc., Eugene, OR) antibodies were applied for 2 h at room temperature or overnight at 4°C. To stain nuclei, 4,6- diamidino-2-phenylindole (Sigma-Aldrich) or SYTO13 (1:1000 dilution; Molecular Probes) was used. Samples were mounted in 2.5% 1,4-diazabicyclo [2.2.2] octane (Sigma-Aldrich) and imaged using a Zeiss 710 inverted confocal microscope. The total fluorescence intensity was determined based on signal threshold using ImageJ (Shihan et al., 2021).

### 2.4 Fluorescence-activated cell sorting (FACS)

The right and left male gonads of *Dnd1* ^*+/+*^ *: Oct4-EGFP* and *Dnd1* ^*Ter/+*^ *: Oct4-EGFP* were dissected at E13.5 (normoxia) and E14.5 (normoxia and after exposure to hypoxia). The gonads were separated from the mesonephros, and individually collected in PBS on ice. Germ cells were sorted by FACS as described previously (Jameson et al., 2012), EGFP-positive cells (GFP+) were collected in PBS and centrifuged at 3000 rpm for 5 min at 4°C to pellet the cells. The PBS was aspirated, and cells were used for single cell immunofluorescence or RNA extraction.

### 2.5 RNA extraction and Quantitative-PCR

Using a TRIzol/isopropanol precipitation method, total RNA was extracted from FACS-based GFP + cells collected from seven individual left and right gonads dissected at E13.5 (normoxia) and at E14.5 (normoxia and hypoxia). DNase (18,068–015; Invitrogen) treatment was performed, and cDNA synthesis was carried out using the iScript kit (170–889; Bio-Rad) and 500 ng of total RNA. Quantitative RT-PCR (qPCR) was used to determine relative expression levels of transcripts in the right and left gonads of different genotypes using the StepOnePlus real-time PCR system (Applied Biosystems). Each analysis was performed in three technical replicates. The CT values for genes expressed in germ cells and Sertoli cells were calculated using the germ cell-specific gene *Ddx4* or the Sertoli cell-specific gene *Sox9* as internal controls. The sequences of primers used in this study can be found in Supplementary Table S2.

### 2.6 Single cell pSMAD2 immunofluorescence

Isolated male germ cells were allowed to attach for 20 min to alcian-blue-pretreated slides (Blackburn et al., 2017) in a cold chamber. Subsequently, the cells were fixed for 10 min with ice-cold 2% PFA. The cells were permeabilized with 0.1% Triton X-100. After two washes in PBS, samples were processed for immunofluorescence as described previously. The pSMAD2 antibody (see Supplementary Table S1) was diluted in blocking solution and incubated with samples overnight at 4°C. After three washes in washing solution fluorescent Cy3-conjugated (1:500 dilution; Jackson ImmunoResearch) secondary antibody was applied for 2 h at room temperature. To stain nuclei, Hoechst 33342 (Invitrogen) was used. Samples were imaged with a Zeiss 710 confocal microscope. The images were processed with ImageJ (Schneider et al., 2012) to determine the *Corrected Total Cell Fluorescence* (CTCF) of single cells using the following formula CTCF = Integrated Density—(Area of selected cell * mean fluorescence of background readings).

### 2.7 Statistical analysis

All data are presented as mean +SEM. The data shown represent the average of at least three independent experiments (please refer to figure legend for exact n). Statistical analysis was performed using GraphPad Prism software (GraphPad Software Inc.), and *p* < 0.05 was considered significantly different. Comparisons were conducted between the right and left gonads, where the right gonad was considered the control sample, therefore measurement in the control gonads were plotted to the left. Differences between gonads or cells exposed to two different experimental conditions were analyzed using Student’s *t* Test. Differences between groups were analyzed using one-way ANOVA, followed by Tukey *post hoc* test.

## 3 Results

### 3.1 Incidence of testicular teratoma in 129 Sv/J *Dnd1* ^*Ter/+*^ mice is twice as high in the left testis

In the breeding pool used for these experiments, the 129 Sv/J *Dnd1* ^*Ter/Ter*^ (homozygous) mice showed ∼64% incidence of testicular teratomas, and 40% of the cases were bilateral tumors. In a cohort of 605 *Dnd1* ^*Ter/+*^ (heterozygous) mice screened, ∼16% of the mice developed testicular teratoma. Of these, ∼12% of the cases were unilateral tumors with a 68% occurrence in the left testis (Table 1). Interestingly, the left-bias in tumor incidence was observed in wild type mice (*Dnd1* ^*+/+*^ littermates) where the total tumor incidence was ∼2%, and in 80% of these cases teratomas developed in the left testis (Table 1).

**TABLE 1:** Incidence of testicular teratoma in 129 Sv/J *Dnd1* ^*Ter*^ mice.

Phenotype	*Dnd1* ^*+/+*^	*Dnd1* ^*Ter/+*^	*Dnd1* ^*Ter/Ter*^
Tumor Incidence	2.4%	16.1%	63.5%
Bilateral tumor	2/420 (0.5%)	14/605 (3.3%)	78/193 (40.4%)
Unilateral teratoma	8/420 (1.9%)	75/605 (12.4%)	43/193 (22.3%)
Right teratoma	2/8 (20%)	24/75 (32%)	18/43 (41.9%)
Left teratoma	6/8 (80%)	51/75 (68%)	25/43 (58.1%)

### 3.2 A model to study the effect of low oxygen on the incidence of testicular teratoma in 129 Sv/J *Dnd1* ^*Ter/+*^ mice

**FIGURE 1:**
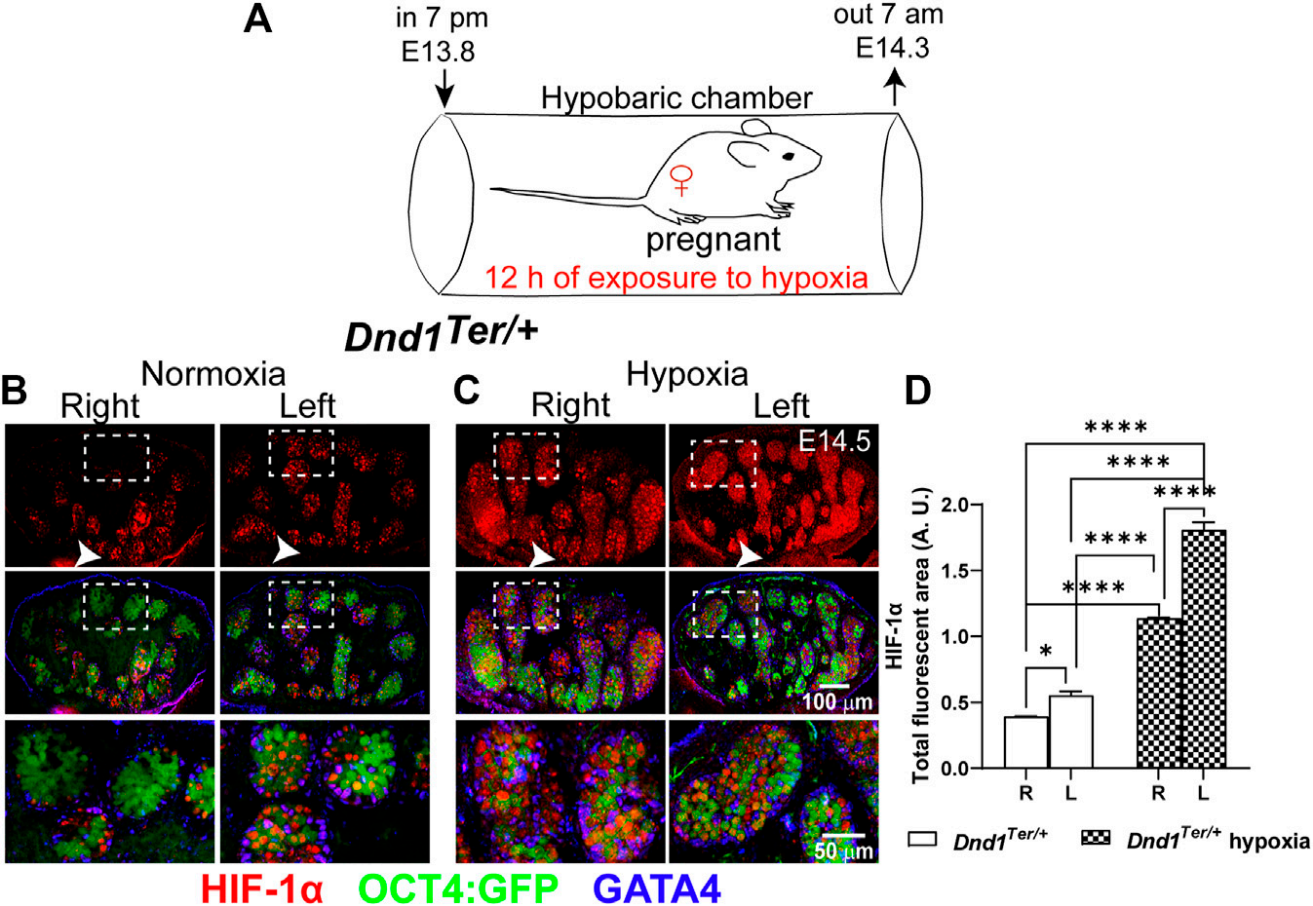
Low systemic oxygen induces HIF-1α in male gonads. **(A)** Experimental design to induce hypoxia *in vivo.* Pregnant 129 Sv/J *Dnd1* ^*Ter/+*^ *:Oct4:GFP* females were exposed to 12 h of hypoxia (10% oxygen) between E13.8 and E14.3. Subsequently, male gonads were dissected at E14.5. Evaluation of HIF-1α by immunofluorescence in **(B)** normoxia (n = 5 embryos), the left gonads have more HIF-1α positive (red) Oct4:GFP positive (green) germ cells compared to the right gonad, in which HIF-1α positive cells were often localized near the rete testis (arrow head) and **(C)** After exposure to hypoxia (n = 5 embryos), HIF-1α, showed a wide distribution in germ cells and supporting cells (GATA4, blue) in all the regions of the left and right gonad. **(D)** Analysis of the total fluorescence intensity was determined based on signal threshold. Statistical significance between the right and left gonads were determined using one way anova multicomparison test with Tukey’s correction. *p*-values are represented by asterisk where *: *p* < 0.05, **: *p* < 0.01, ***: *p* < 0.001, and ****: *p* < 0.0001.

Our previous studies of male germ cell survival in 129 Sv/J *Dnd1* ^*Ter/+*^ mice showed increased levels of HIF-1α in the left testis compared to the right testis. High levels of HIF-1α in the left testis strongly correlated with anatomical differences in vascular architecture between the right and left testes (Bustamante-Marin et al., 2015). We hypothesized that *Dnd1* ^*Ter/+*^ SSCs are sensitized to environmental challenges that can influence their fate (Bustamante-Marin et al., 2015). To investigate the role that oxygen levels play during the commitment of PGCs to the male germ cell pathway, we developed a method to induce a systemic reduction of oxygen by maintaining pregnant females in a hypobaric chamber. The hypobaric chamber was pressurized at ∼ 53 kPa, reducing the availability of oxygen to ∼10%. Under these conditions, we calculated that the partial pressure of arterial oxygen was ∼3.5 kPa. To test the system, pregnant females at E13.8 were maintained in the hypobaric chamber for 12 h, between 7 a.m. and 7 p.m. (Figure 1A). To confirm that we simulated a low oxygen microenvironment, male left and right gonads were dissected at E14.5 and immunofluorescence was used to detect HIF-1α positive cells in these gonads compared to normoxic controls (Figure 1). Under normoxic conditions, as expected, the left gonad had more HIF-1α positive cells compared to the right gonad (Figures 1B, D). Under normoxic conditions, germ cells that showed high levels of HIF-1α localized near the rete testis (Figure 1B, arrow). In experimental samples that experienced a 50% reduction of atmospheric oxygen availability (Figures 1C, D), high levels of HIF-1α were stabilized in multiple seminiferous cords of both left and right male gonads. Thus, these results show that our method for inducing systemic hypoxia resulted in a widespread expression of HIF-1 α in male gonads.

### 3.3 Low oxygen availability, during a specific window of germ cell development, increases the incidence of testicular teratoma

**FIGURE 2:**
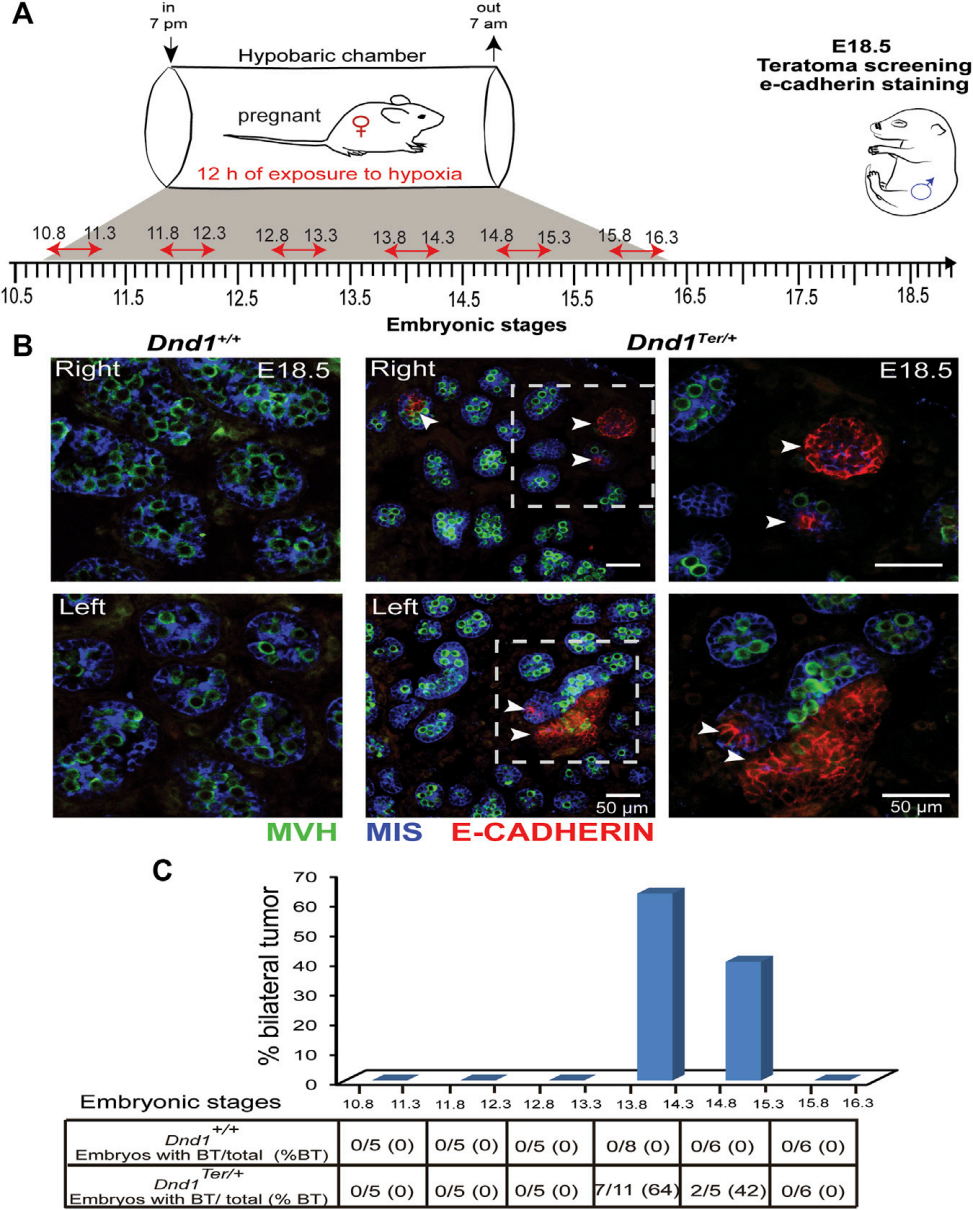
Hypoxia increases the incidence of testicular teratoma. **(A)** 129/SvJ *Dnd1* ^*Ter/+*^ pregnant females, at different embryonic stages between E10.8 and E15.8*,* were maintained for 12 h (indicated by the red arrow) under hypoxic conditions (10% oxygen). After exposure to hypoxia, the pregnant females were transferred back to normoxic conditions until gonads were dissected from male fetuses at E18.5 for **(B)** Representative screening for incidence of testicular teratoma by immunofluorescence at E18.5. In this example, a pregnant female was exposed to hypoxia between E13.8 and E14.3. The right and left gonads of wild type (left panel) and *Dnd1* ^*Ter/+*^ (middle panel) were labeled with MVH (germ cell, green), MIS (Sertoli cells, blue) and E-Cadherin (tumorigenic cell, red). Clusters of E-Cadherin positive cells were found bilaterally in *Dnd1* ^*Ter/+*^ testes. **(C)** Percentage of bilateral teratoma (%BT) incidence. In wild type gonads, no E-Cadherin cluster was detected at any time point of exposure to hypoxia. In *Dnd1* ^*Ter/+*^ gonads, the exposure to hypoxia, between E13.8 to 14.3 and E14.8 to 15.3, significantly increased the incidence of bilateral testicular teratomas. The number (#) of embryos with bilateral tumor over the total number of embryos screened (Embryos with BT/total) is indicated in the table in **(C)**.

Based on our finding that exposure of pregnant dams to hypoxic conditions stabilized HIF-1α in both right and left gonads, we hypothesized that the systemic reduction of oxygen levels, would abolish the left-bias in teratoma incidence in 129/SvJ *Dnd1* ^*Ter/+*^ mice*.* We speculated that male germ cells are more sensitive to hypoxia exposure during specific windows of development prior to the time when teratomas are detected (E15.5-E16.5). To test this hypothesis, we exposed pregnant females to hypoxic conditions for a single period of 12 h, testing each day of development between E10.8 and E15.8 (Figure 2A). After exposure for 12 h to hypoxic conditions, pregnant females were maintained in a normoxic environment, and male gonads were dissected at E18.5. To evaluate the incidence of testicular teratoma, we used immunofluorescence to detect clusters of E-Cadherin positive cells, an early marker of teratoma formation (Cook et al., 2009) (Figure 2B). Our results showed that systemic exposure to hypoxia eliminated the left bias of teratoma development and increased the incidence of E-Cadherin clusters in both gonads (Figure 2B). Interestingly, the increase in bilateral tumors was dependent on the embryonic stage at which the gonads were exposed to hypoxia (Figure 2C). A total of 37 *Dnd1* ^*Ter/+*^ male embryos exposed to hypoxia between E10.8 and E15.8 were screened. Exposure of 129/SvJ *Dnd1* ^*Ter/+*^ male embryos to hypoxia between E10.8 and E12.8 did not increase the incidence of bilateral tumors, although it slightly increased the incidence of unilateral tumors to 13.5% with equal numbers of left and right gonads showing signs of teratoma development by E18.5. In contrast, exposure of 129/SvJ *Dnd1* ^*Ter/+*^ male embryos to 12 h of hypoxia between embryonic stages E13.8—E14.3 and E14.8—E15.4 significantly increased the incidence of bilateral teratomas to 64% and 40%, respectively (Figure 2C). Together these results show that the overall incidence of bilateral testicular teratoma increased from ∼3% under normoxia (as shown in Table 1) to more than 50% after exposure to hypoxia between E13.8 and 15.3 (Figure 2C). These results also suggest that the period of germ cell development, just prior to and during the stage when male germ cells enter mitotic arrest is the most susceptible to teratoma development-induced by low oxygen availability.

### 3.4 Hypoxia maintains the expression of germ cell pluripotency genes

**FIGURE 3:**
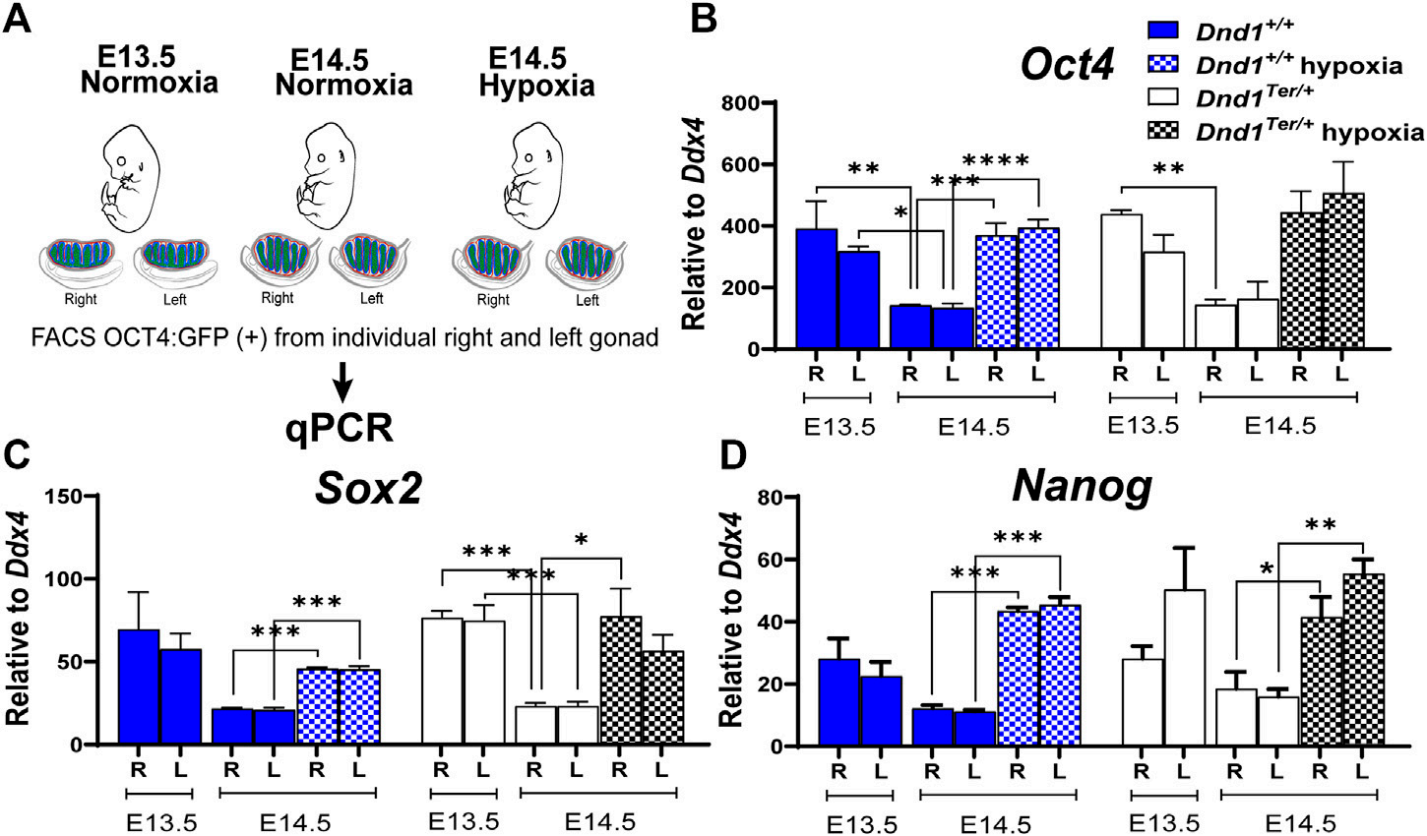
Hypoxia promotes the expression of the core pluripotency genes in germ cells *in vivo*. **(A)** Schematic representation of the experimental design. Pregnant females were exposed to hypoxia between E13.8 and E14.3. The hypoxic right (R) and left (L) gonads, toguether with normoxic controls, were disected at E13.5 and E14.5. The OCT4:GFP(+) cells were sorted by FACS and total RNA was extracted to perform qPCR evaluating the expression of **(B)** *Oct4* (*Dnd1* ^*+/+*^ and *Dnd1* ^*Ter/+*^ E13.5 and E14.5 n = 3; *Dnd1* ^*+/+*^ and *Dnd1* ^*Ter/+*^ hypoxia n = 5) **(C)** *Sox2* (*Dnd1* ^*+/+*^ and *Dnd1* ^*Ter/+*^ E13.5 and E14.5 n = 3; *Dnd1* ^*+/+*^ and *Dnd1* ^*Ter/+*^ hypoxia n = 3) and **(D)** *Nanog* (*Dnd1* ^*+/+*^ and *Dnd1* ^*Ter/+*^ E13.5 and E14.5 n = 3; *Dnd1* ^*+/+*^ and *Dnd1* ^*Ter/+*^ hypoxia n = 3). Gene expression was normalized to *Ddx4*. Statistical significance between the gene expression in germ cells from the right or left gonads at different ebryonic stage or oxygen levels were determined using one way ANOVA multicomparison test with Tukey’s correction. *p*-values are represented by asterisk where *: *p* < 0.05, **: *p* < 0.01, ***: *p* < 0.001, and ****: *p* < 0.0001.

When germ cells arrive in the gonad, they express the core pluripotency genes, *Nanog*, *Sox2* and *Oct4*, similar to embryonic stem cells. At this stage they are capable of giving rise to germline stem cells or spontaneously forming teratomas. Downregulation of these genes occurs as male germ cells commit to the male pathway and enter mitotic arrest and is associated with a sharp decline in the tendency to form teratomas. At E12.5 ∼ 60% of germ cells can be found in S or G2/M phases of the cell cycle; however, by E14.5 most of the germ cells are in G1/G0 (Western et al., 2011). Because pregnant females exposed to hypoxia between E13.8 and E14.3, showed significant increases in bilateral tumors by E18.5, we evaluated the effect of hypoxia on the expression of O*ct4, Sox2,* and *Nanog* at E14.5 and compared them to those measured under normoxia at E13.5 and E14.5 (Figure 3A). As expected, in male germ cells from 129 Sv/J *Dnd1* ^*+/+*^ and *Dnd1* ^*Ter/+*^ mice developed under normoxia, the levels of these genes were elevated at E13.5 and we observed a tendency to be downregulated by E14.5 in both right and left gonads of both genotypes. In contrast, in male germ cells from *Dnd1* ^*+/+*^ and *Dnd1* ^*Ter/+*^, the exposure to acute hypoxia (Figures 3B, C, and D) resulted in the maintenance of high expression of *Oct4*, *Sox2* and *Nanog*. We did not observe significant differences between the expression of these genes in right and left gonads (Supplementary Table S3) To investigate the effect of acute hypoxia on supporting cells of the gonad, we evaluated the expression of three genes characteristic of Sertoli cells, *Fgf9* (Willerton et al., 2004), *Sox9* and *Wt1* in *Oct4:GFP-negative* cells. We detected no changes induced by hypoxia on the expression of these genes (Supplementary Figure S1). These results show that hypoxia promotes the maintenance of pluripotency genes in male germ cells beyond E14.5 in both the right and left gonads of both genotypes. This result is consistent with the idea that reduced oxygen availability is one of the factors underlying the bias of testicular teratoma in the left testis of *Dnd1* ^*Ter/+*^ mice.

### 3.5 The Nodal pathway remains activated in gonads exposed to hypoxia

**FIGURE 4:**
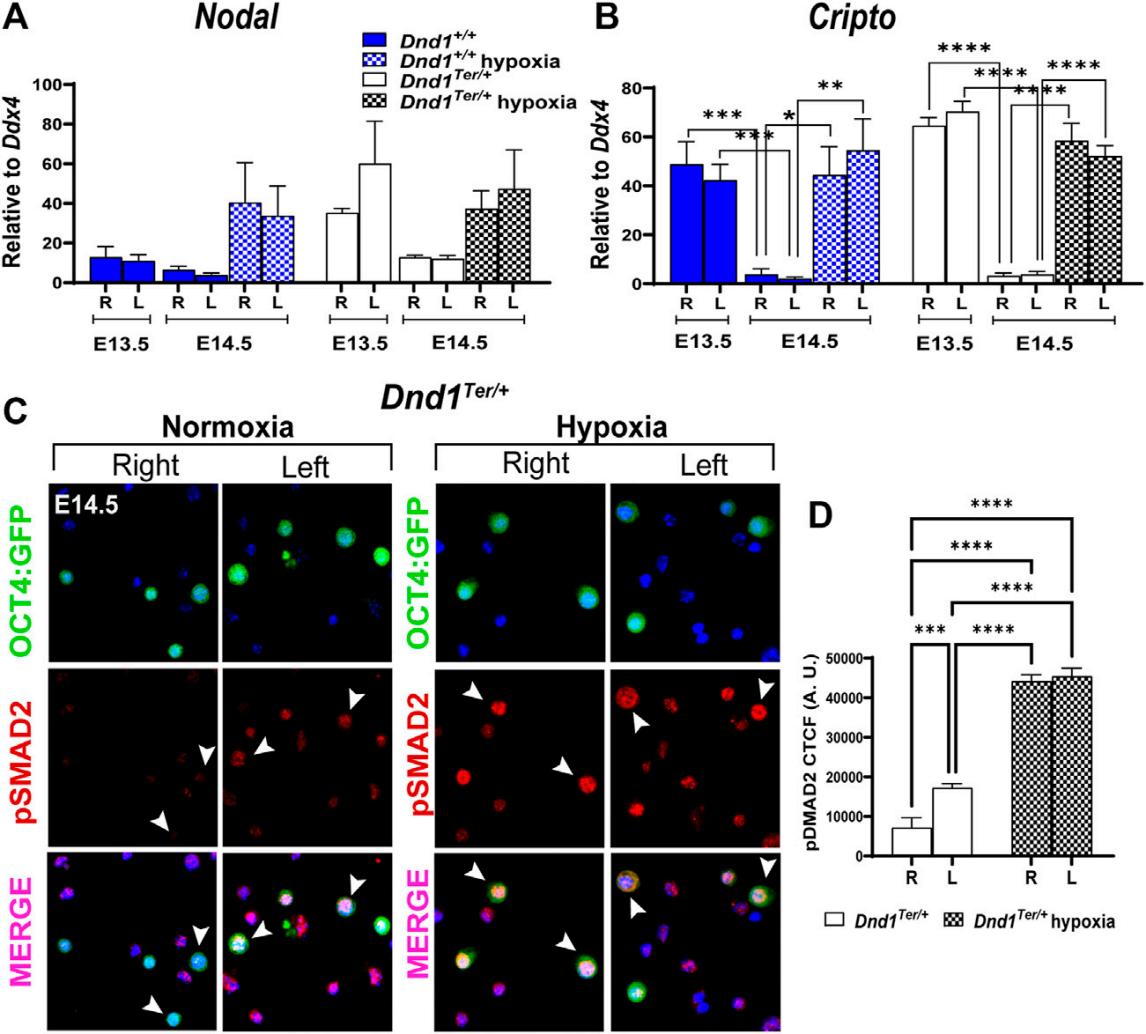
Hypoxia promotes the activation of the Nodal signal pathway. Pregnant females were exposed to hypoxia between E13.8 and E14.3. The hypoxic right (R) and left (L) gonads, together with normoxic controls, were dissected at E13.5 and E14.5. The OCT4:GFP(+) germ cells were sorted by FACS and total RNA was extracted to perform qPCR evaluating the expression of **(A)** *Nodal* and **(B)** *Cripto* (*Dnd1* ^*+/+*^ and *Dnd1* ^*Ter/+*^ at E13.5 n = 9; *Dnd1* ^*+/+*^ and *Dnd1* ^*Ter/+*^ at E14.5 normoxia n = 5; *Dnd1* ^*+/+*^ and *Dnd1* ^*Ter/+*^ at E14.5 hypoxia n = 8). Gene expression was normalized to *Ddx4*. Statistical significance between groups of right and left gonads of different embryonic stage or oxygen levels were determined using one way ANOVA multicomparison test with Tukey’s correction. **(C)** Immunostaining of isolated germ cells (OCT4:GFP(+)). Germ cells isolated from the right and left gonads were attached to a slide (see methods) and stained using an antibody against phospho-SMAD2 (red). The nuclear staining was conducted using Hoechst 33,342 (blue). Arrowheads show double labeled OCT4:GFP+ and pSMAD2+ cells. Representative images of n = 3 experiments. **(D)** Corrected total cell fluorescence (CTCF) of OCT4:GFP + cells positive for pSMAD2 is elevated in germ cells isolated from the left and right testis after embryonic exposure to hypoxia.

The Nodal signaling pathway governs stem cell function during cell fate decisions, organogenesis, and homeostasis of adult tissues through the maintenance of expression of pluripotency genes in human and mouse stem cells (Vallier et al., 2004; Brons et al., 2007) and by crosstalk with cell cycle pathways (Pauklin and Vallier, 2013). In mouse male germ cells, the Nodal co-receptor, *Cripto*, is expressed transiently at E12.5, preceding the spike of *Nodal* at E13.5 (Bustamante-Marin et al., 2015; Spiller et al., 2017). However, under normoxic conditions, the expression of both genes is rapidly downregulated. To study the effect of oxygen availability on Nodal signaling, we evaluated the expression of *Nodal* and *Cripto* in germ cells sorted by FACS from right and left gonads developing in normoxic or hypoxic conditions. As expected, under normoxia in male germ cells from 129 Sv/J *Dnd1* ^*+/+*^ and *Dnd1* ^*Ter/+*^ mice, the levels of *Nodal* and *Cripto* were elevated at E13.5 in germ cells from each gonad and significantly decreased by E14.5 (Figures 4A,B), however, the differences were not statistically different for *Nodal*. On the other hand, the downregulation of *Cripto* was statistically different between the right or left gonads at different stages of embryonic development. However, in germ cells exposed to hypoxia between E13.8—E14.3, the levels of *Nodal* and *Cripto* remained elevated at E14.5 (Figures 4A,B). We also measured the expression levels of the negative regulators of Nodal, *Lefty1* and *lefty2* (Supplementary Figure S2). We observed a decrease in the levels of these genes at E14.5 compared to E 135.5. However, hypoxia differentially affected the levels of these genes which were increased for *Lefty1* and remained low for *Lefty2*.

The high levels of *Nodal* and *Cripto* expression correlated with high activation of the downstream effector of the Nodal pathway, SMAD2 (Figure 4C). We observed high levels of phospho-SMAD2 (pSMAD2) in germ cells in the left gonad compared to the right (Figures 4C,D) and the levels increased after the exposure to hypoxia (Figures 4C,D). These results show that hypoxia prolongs Nodal signaling and the activation of pSMAD2, which might contribute to the miss-regulation of the core pluripotency genes and to the development of testicular teratoma.

### 3.6 Hypoxia stimulates active cell cycle in male gonads

**FIGURE 5:**
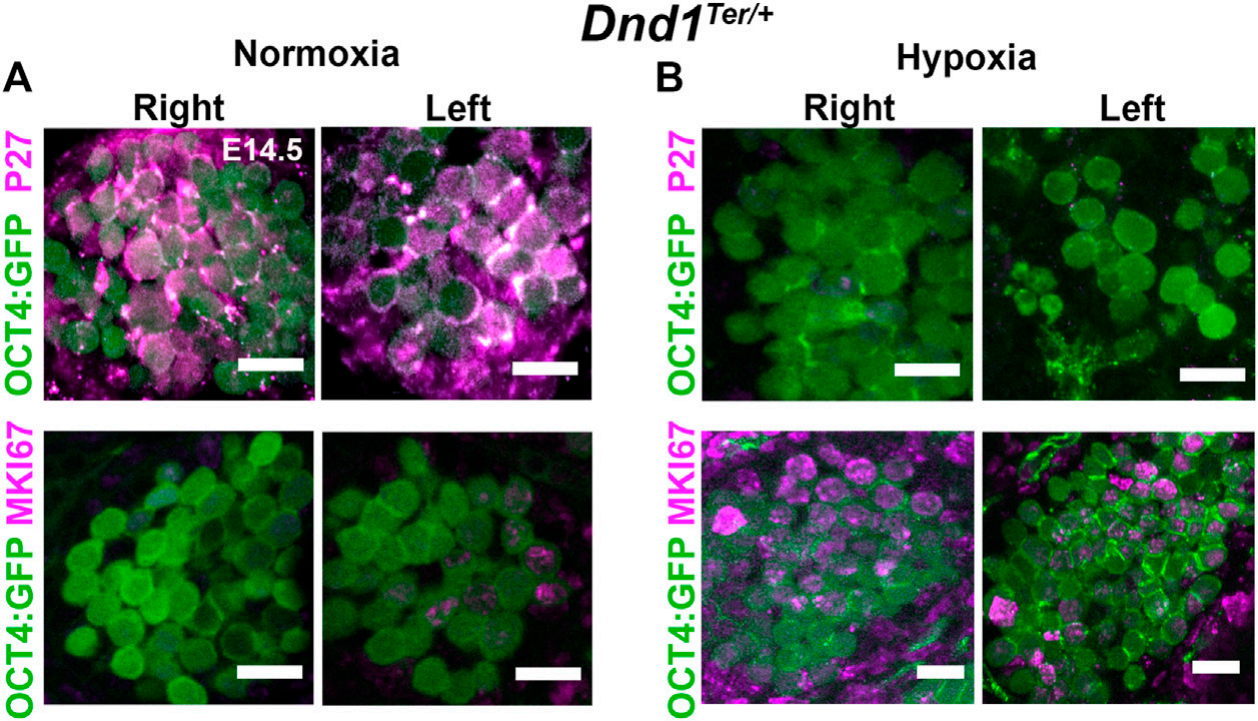
Exposure to hypoxia stimulates the cell cycle in germ cells. Pregnant females were exposed to hypoxia between E13.8 and E14.3. The hypoxic right (R) and left (L) gonads, together with normoxic controls, were disected at E14.5. *Dnd1* ^*Ter/+*^ *:Oct4:GFP* gonads where stained for MKI67 (an active cell cycle marker, magenta) or P27 (negative regulator of the cell cycle, magenta). **(A)** Under normoxic conditions, germ cells (OCT4+, green) enter G0/G1 arrest (p27 positive; MKI67 negative) **(B)** After hypoxic exposure, most germ cells (green) remain in active cell cycle at E14.5 (P27 negative; MKI67 positive, red). Representative image of n = 3 experiments. Scale bar 10 µm.

During germ cell development, failure to repress pluripotency and enter cell cycle arrest may render germ cells susceptible to tumorigenic transformation. In mice, the entry of male germ cells into cell cycle arrest in G0 is controlled by strict regulation of the proteins involved in the G1/S transition and is detected by the downregulation of MKI67, a cellular marker for all active phases of the cell cycle, and up-regulation of p27, a negative regulator of the cell cycle (Western et al., 2008; Cook et al., 2011). To investigate the effect of hypoxia on mitotic arrest, we evaluated the abundance of p27 and MKI67 (Figure 5). Under normoxia, most of the germ cells in the right and left testis were positive for p27 and negative for MKI67 (Figure 5A). In contrast, gonads exposed to acute hypoxia maintained an active cell cycle (MKI67 + cells), while p27 was barely detectable (Figure 5B). Although many aspects of the pathway to mitotic arrest in male germ cells have still to be genetically determined, these results suggest that hypoxia prolongs the active cell cycle in male germ cells.

## 4 Discussion

These results show that the gonad microenvironment during a window of germ cell development between E13.8 and E14.8 is critical for the preservation of the germ cell lineage commitment in 129/SvJ *Dnd1* ^*Ter/+*^ mice. Male germ cells exposed to hypoxia fail to downregulate pluripotency genes, suppress Nodal/Smad signaling, and undergo cell cycle arrest. All of these factors are associated with promotion of testicular teratomas (Cook et al., 2011; Heaney et al., 2012; Spiller et al., 2012; Webster et al., 2021).

Our previous work showed that differences in vascular architecture between the left and right testis of adult 129/SvJ mice lead to lower oxygen pressure and elevation of HIF-1α in the left testis (Bustamante-Marin et al., 2015). These differences correlated with the high incidence of testicular failure (95%) in the left testis of 129/SvJ *Dnd1* ^*Ter/+*^ mice. Additionally, adult 129/SvJ *Dnd1* ^*Ter/+*^ mice have a 12% incidence of unilateral testicular teratoma, and 68% of the cases occur in the left testis. Although the origin of testicular teratomas is not completely understood, they may arise from germ cell neoplasia *in situ* (GCNIS) cells that originate from the neoplastic transformation of SSCs or of latent PGCs, as it has been suggested that PGCs that fail to transition to SSCs can persist in a dormant state and later give rise to tumors (Rajpert-De Meyts et al., 2000). The teratoma phenotype in 129/SvJ *Dnd1* ^*Ter/+*^ mice opens the possibility of investigating mechanisms that leads to the misregulation of germ cell differentiation. We hypothesized that the left bias in testicular teratoma development was due to differences in oxygen tension between the right and left testis. Hypoxia promotes the undifferentiated cell state in various stem cell populations. For example, in neurons, hypoxia blocks differentiation in a Notch-dependent manner. The Notch intracellular domain interacts with HIF-1α, activating the Notch-responsive promoters and increasing expression of Notch downstream genes (Gustafsson et al., 2005). In mesenchymal stem cells (MSCs), low oxygen tension is an important factor in the determination of cell fate and maintenance of stemness in adipose and bone marrow-derived MSCs (Fotia et al., 2015; Kim et al., 2016; Mas-Bargues et al., 2019). Recently the testis microenvironment has been proposed to regulate mitochondrial function and oxidative phosphorylation in spermatogonia (Lord and Nixon, 2020). Our results present a possible mechanism for the effects of hypoxia on male germ cell pluripotency. 1) Low oxygen tension preserves germ cell stemness by promoting the expression of *Oct4*, *Sox2,* and *Nanog*. This is supported by recent results suggesting that reduced oxygen tension was the major factor improving the regenerative capacity of SSCs (Helsel et al., 2017) and by the finding that hypoxia promotes re-entry of committed cells into pluripotency by inducing the re-activation of the *OCT4*-promoter (Mathieu et al., 2013). 2) Low oxygen tension maintains active Nodal/Smad2 signaling. In male germ cells and somatic cells, the re-activation of the Nodal/Smad2 pathway has been related to malignant transformation (Postovit et al., 2007; Spiller et al., 2012). In support of this connection, Nodal induced by hypoxia contributes to the maintenance of stemness in melanoma cancer cells (Li et al., 2018). Finally, 3) Hypoxia suppresses the entry of germ cells into mitotic arrest.

The association among failure to downregulate pluripotency genes, the elevation of Nodal/Smad2 signaling, and failure to undergo cell cycle arrest has been reported by multiple investigators studying *Dnd* mutants (Cook et al., 2011; Heaney et al., 2012; Yamaji et al., 2017; Ruthig et al., 2019; Ruthig et al., 2021), and other models of germ cell tumor development (Spiller et al., 2012; Schemmer et al., 2013). Among this trifecta of factors, it is not clear which is the primary driver of the transition of germ cells to the teratoma/tumor fate. However, it has often been suggested that cell cycle control of male germ cells is required for their genetic reprogramming and commitment to the SSC lineage (Western, 2009; Cook et al., 2011; Heaney et al., 2012; Ruthig et al., 2019).

While the failure to undergo cell cycle arrest is associated with tumor formation by definition, its role in the process might be more causative. In male fetal germ cells, arrest in G1/G0 occurs at E 14.5-E15.5 and persists until after birth. Prior to this stage, teratomas spontaneously arise and can be induced by transplantation of the testis to the kidney capsule of a host mouse (Stevens, 1964; Bustamante-Marin et al., 2013). This may explain the stage-specific effects of hypoxic exposure in this study. By E15.5, the ability to induce teratomas declines, suggesting that during cell cycle arrest, genomic and epigenetic changes occur that limit tumor susceptibility. Although this period of germ cell arrest is not well characterized, epigenetic modifications of the germ cell genome occur which lead to the postnatal emergence of a stabilized SSC population with a (usually) low propensity to form tumors (Seisenberger et al., 2012; Kubo et al., 2015). Exactly how this occurs is a critical question in male germ cell biology, but it could reveal that G0 arrest is critical to protect male germ cells from spontaneously embarking on a tumor pathway. Factors that prevent cell cycle arrest and drive proliferation might play a causative role in tumor formation.

The maintenance of expression of pluripotency factors occurs in various mutants with defects in *Dnd1*. DND1 binds to transcripts associated with pluripotency and, cooperatively with NANOS2, recruits them to the CCR4-NOT complex for degradation (Suzuki et al., 2016; Yamaji et al., 2017; Ruthig et al., 2019). While this function is likely compromised in the heterozygous *Dnd1* ^*Ter/+*^ mutants, it seems probable that the elevation of pluripotency genes has an additional contributor, the Nodal/Smad2 pathway. Smad proteins act as transcriptional regulators that mediate the crosstalk between the Activin/Nodal and BMP signaling pathways (Luo, 2017). While BMP signals seem to lead to the differentiation of germ cells (Dudley et al., 2010), Nodal signaling is associated with the establishment/maintenance of pluripotency (Spiller et al., 2012; Spiller et al., 2017; Jørgensen et al., 2018). Smads can induce or repress the transcription of their target loci by recruiting epigenetic modifiers, which modulate the accessibility of the surrounding chromatin by inducing epigenetic modifications of histones or DNA. Epigenetic regulation of germ cell fate has been demonstrated to be a regulator of susceptibility to germ cell tumors (Nettersheim and Schorle, 2017; Müller et al., 2022), and it is clear that low oxygen conditions and the associated switch to glycolytic metabolism affect epigenetic regulation (Burr et al., 2017; Lai et al., 2018). To test these possibilities, it would be interesting to cross the 129/SvJ *Dnd1* ^*+/−*^ mice to the hypomorphic *Nodal* ^*flox/flox*^ mouse line (Lowe et al., 2001). The reduction of germ cell pluripotency due to decreased Nodal activation should abolish the response to hypoxia. The Nodal antagonist *Lefty1* and *Lefty2* are other possible candidates to continue investigating the impact of microenvironment on testicular teratoma development. These genes seem to be affected differentially by hypoxia. It has been shown that Lefty and Nodal overexpression promote glioblastoma cells survival under hypoxia (Matsumoto et al., 2020).

The importance of the input from the microenvironment on germ cell differentiation, plasticity and tumor incidence *in vivo* and *in vitro* has been noted previously (Nettersheim and Schorle, 2017) (Xie et al., 2015). Here we show that *Dnd1* ^*Ter/+*^ germ cells are sensitized to their microenvironment and that low oxygen tension, through the activation of Nodal/Smad2 pathway, promotes misregulation of male germ cell development, leading to teratoma initiation. This is a possible mechanism to explain the left-bias of teratoma development in 129/SvJ *Dnd1* ^*+/−*^ mice.

## Data Availability

The original contributions presented in the study are included in the article/Supplementary Material, further inquiries can be directed to the corresponding author.
