# Supplementary material for: Oxygen availability influences the incidence of testicular teratoma in Dnd1Ter/+ mice
Source: Front Genet. 2023 Apr 26;14:1179256. doi: 10.3389/fgene.2023.1179256 (PMC10169730; doi:10.3389/fgene.2023.1179256)
Supplement: Supplementary file 1 [file DataSheet1.docx]

**Supplementary Table 1: Primary Antibodies**

| **Antibody Name** | **host** | **company** | **Catalog#** | **dilution** |
| --- | --- | --- | --- | --- |
| AMH/MIS | goat | Santa Cruz BT | sc-6886 (discontinued) | 1:100 |
| E-Cadherin | rat | Novus Biologicals | 13-1900 | 1:500 |
| GATA4 | goat | Santa Cruz BT | sc-1237 (discontinued) | 1:100 |
| HIF-1α | rabbit | Novus Biologicals | NB100-479 | 1:2000 |
| MKI67 | rabbit | NeoMarkers | RM-9106-S | 1:500 |
| MVH | rabbit | Abcam | ab13840 | 1:500 |
| P-SMAD2 | rabbit | Cell Signaling | 3101 | 1:500 |
| P27 | rabbit | Santa Cruz BT | sc528 | 1:100 |
| P27 | rabbit | Cell Signaling | 3688 | 1:1000 |
| SOX9 | rabbit | Millipore | ab5535 | 1:2000 |

**Supplementary Table 2: Primers used in this study**

| **Gene Symbol** | **Forward primer 5’ -> 3’** | **Reverse Primer 5’ -> 3’** |
| --- | --- | --- |
| *Cripto* | CGTCGAAGATGGGGTACTTC | ACGGGTCCAAATTCAAACGC |
| *Ddx4* | TCAGGAACTGTCAGACGCTCA | ATTCAACGTGTGCTTGCCCT |
| *Fgf9* | CAGGGAACCAGGAAAGACCA | GAGGTAGAGTCCACTGTCCAC |
| *Gapdh* | AGGTCGGTGTGAACGGATTTG | TGTAGACCATGTAGTTGAGGTCA |
| *Nodal* | CATGTTGAGCCTCTACCGAGAC | CGTGAAAGTCCAGTTCTGTCCG |
| *Nanog* | TGAGCTATAAGCAGGTTAAGAC | CAATGGATGCTGGGATACTC |
| *Oct4* | GGAGGAAGCCGACAACAATGA | TCCACCTCACACGGTTCTCAA |
| *Sox2* | TGGACTGCGAACTGGAGAAGG | TGGGTTAATTTGGATGGGATTGG |
| Sox9 | TCCAGCAAGAACAAGCCACAC | TCTCGTTCAGCAGCCTCCAG |
| *Wt1* | GTGTGACTTCAAGGACTGCGA | TGGTGTGGGTCTTCAGATGGT |

**Supplementary Table 3: P-values of Right vs Left gonads comparison of gene expression in Figure 3 and Figure 4 using Student’s t Test.**

| **Genotype** | **E. Stage** | **Condition** | ***Oct4*** | ***Sox2*** | ***Nanog*** | ***Nodal*** | ***Cripto*** |
| --- | --- | --- | --- | --- | --- | --- | --- |
| *Dnd1^+/+^* | E13.5 | Normoxia | 0.5024 | 0.6619 | 0.5296 | 0.7682 | 0.5811 |
| *Dnd1^+/+^* | E14.5 | Normoxia | 0.6142 | 0.6870 | 0.4688 | 0.2777 | 0.5123 |
| *Dnd1^+/+^* | E14.5 | Hypoxia | 0.6280 | 0.8571 | 0.5337 | 0.7940 | 0.5740 |
| *Dnd1^Ter/+^* | E13.5 | Normoxia | 0.1508 | 0.8791 | 0.2353 | 0.3013 | 0.3074 |
| *Dnd1^Ter/+^* | E14.5 | Normoxia | 0.7699 | 0.9775 | 0.6918 | 0.7499 | 0.7507 |
| *Dnd1^Ter/+^* | E14.5 | Hypoxia | 0.6257 | 0.5945 | 0.1638 | 0.6563 | 0.4729 |

**
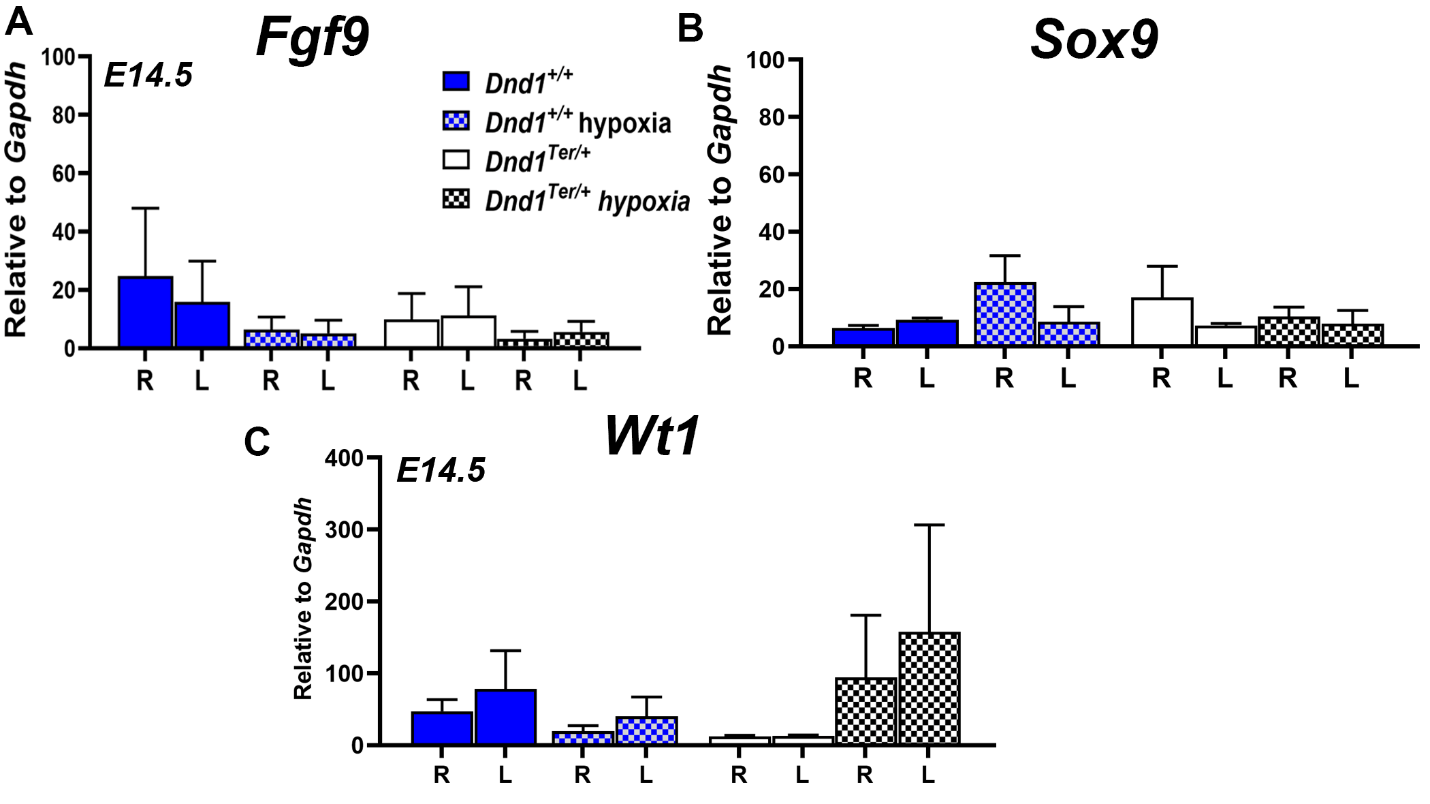
**

**Supplementary Figure 1: Hypoxia does not affect the expression of supporting cells-associated genes.** Pregnant females were exposed to hypoxia between E13.8 and E14.3. The hypoxic right (R) and left (L) gonads, together with normoxic controls, were dissected at E14.5. The OCT4:GFP(-) cells were sorted and total RNA was extracted to perform qPCR evaluating the expression of **A**) *Fgf9* and **B**) *Sox9* and **C**) *Wt1.* The gene expression was normalized to *Gapdh.*  No statistical significance between groups of right and left gonads under different oxygen levels was determined using one way ANOVA multicoparison test with Tukey’s correction, n = 3 for each gonad/condition.


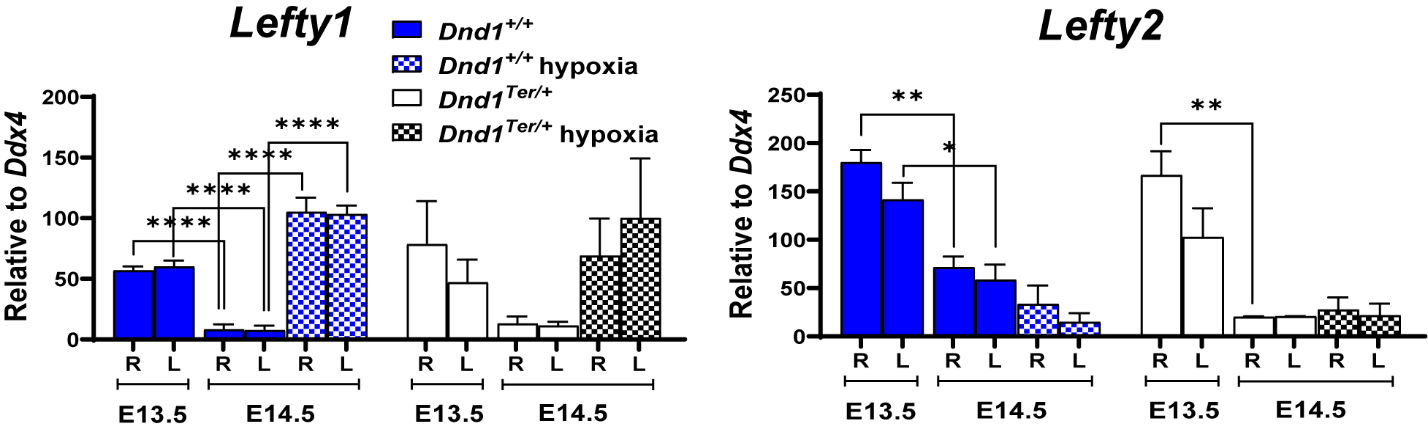


**Supplementary Figure 2: Hypoxia differentially affect the expression of *Lefty1* and *Lefty2*. supporting cells-associated genes.** Pregnant females were exposed to hypoxia between E13.8 and E14.3. The hypoxic right (R) and left (L) gonads, together with normoxic controls, were dissected at E13.5 and E14.5. The OCT4:GFP(+) germ cells were sorted by FACS and total RNA was extracted to perform qPCR evaluating the expression of *Left1* and *Lefty2* (at E13.5 for *Lefty 1* *Dnd1^+/+^* n = 4 and *Dnd1^Ter/+^* n = 6; for *Lefty 2* *Dnd1^+/+^* n = 3 and *Dnd1^Ter/+^* n = 4. At E14.5 normoxia for *Lefty1* *Dnd1^+/+^* n = 5 and *Dnd1^Ter/+^* n = 3; for *Lefty2* *Dnd1^+/+^* n = 3 and *Dnd1^Ter/+^* n = 3. At E14.5 hypoxia for *Lefty1 and Lefty2* *Dnd1^+/+^* n = 3 and *Dnd1^Ter/+^* n = 5). Gene expression was normalized to *Ddx4*. Statistical significance between groups of right and left gonads of different embryonic stage or oxygen levels were determined using one way ANOVA multicomparison test with Tukey's correction
